# Molecular Cytogenetic Identification of a New Wheat-Rye 6R Chromosome Disomic Addition Line with Powdery Mildew Resistance

**DOI:** 10.1371/journal.pone.0134534

**Published:** 2015-08-03

**Authors:** Diaoguo An, Qi Zheng, Qiaoling Luo, Pengtao Ma, Hongxia Zhang, Lihui Li, Fangpu Han, Hongxing Xu, Yunfeng Xu, Xiaotian Zhang, Yilin Zhou

**Affiliations:** 1 Center for Agricultural Resources Research, Institute of Genetics and Developmental Biology, Chinese Academy of Sciences, Shijiazhuang, China; 2 The State Key Laboratory of Plant Cell and Chromosome Engineering, Institute of Genetics and Developmental Biology, Chinese Academy of Sciences, Beijing, China; 3 The National Key Facility for Crop Gene Resources and Genetic Improvement, Institute of Crop Science, Chinese Academy of Agricultural Sciences, Beijing, China; 4 The State Key Laboratory for Biology of Plant Disease and Insect Pests, Institute of Plant Protection, Chinese Academy of Agricultural Sciences, Beijing, China; Julius Kuehn-Institute (JKI), GERMANY

## Abstract

Rye (*Secale cereale* L.) possesses many valuable genes that can be used for improving disease resistance, yield and environment adaptation of wheat (*Triticum aestivum* L.). However, the documented resistance stocks derived from rye is faced severe challenge due to the variation of virulent isolates in the pathogen populations. Therefore, it is necessary to develop desirable germplasm and search for novel resistance gene sources against constantly accumulated variation of the virulent isolates. In the present study, a new wheat-rye line designated as WR49-1 was produced through distant hybridization and chromosome engineering protocols between common wheat cultivar Xiaoyan 6 and rye cultivar German White. Using sequential GISH (genomic *in situ* hybridization), mc-FISH (multicolor fluorescence *in situ* hybridization), mc-GISH (multicolor GISH) and EST (expressed sequence tag)-based marker analysis, WR49-1 was proved to be a new wheat-rye 6R disomic addition line. As expected, WR49-1 showed high levels of resistance to wheat powdery mildew (*Blumeria graminis* f. sp. *tritici*, *Bgt*) pathogens prevalent in China at the adult growth stage and 19 of 23 *Bgt* isolates tested at the seedling stage. According to its reaction pattern to different *Bgt* isolates, WR49-1 may possess new resistance gene(s) for powdery mildew, which differed from the documented powdery mildew gene, including *Pm20* on chromosome arm 6RL of rye. Additionally, WR49-1 was cytologically stable, had improved agronomic characteristics and therefore could serve as an important bridge for wheat breeding and chromosome engineering.

## Introduction

Powdery mildew caused by *Blumeria graminis* f. sp. *tritici* (*Bgt*) is one of the major serious diseases threatening the production of wheat (*Triticum aestivum* L.) in many regions around the world. Although fungicides controlling the disease are available, host resistance has proved to be the most economical and environmentally safe approach to reduce yield losses. However, as co-evolution of pathogen virulence and host resistance, cultivars carrying a single isolate-specific resistance gene, successively lost resistance to pathogens a few years after extensive growing [1]. In recent years, powdery mildew was prevalent in the main wheat producing regions of China and resulted in serious yield loss [2]. To combat the disease, a continuing challenge is faced to discover new gene sources for powdery mildew resistance and incorporate these genes into wheat breeding programs.

Rye (*Secale cereale* L., 2n = 2x = 14, RR), a close relative of common wheat, has already been proved to be a desirable donor of valuable genes for wheat improvement [3]. The development and utilization of the wheat-rye T1RS·1BL and T1RS·1AL translocations are the most successful examples [4]. There were several disease resistant genes located on rye chromosome arms, for instance, the powdery mildew resistance (*Pm*) gene *Pm8*, stripe rust (*Puccinia striiformis* Westend. f. sp. *tritici*) resistance gene *Yr9*, stem rust (*P*. *graminis* Pers. f. sp. *tritici*) resistance gene *Sr31* and leaf rust (*P*. *triticina* Eriks. f. sp. *tritici*) resistance gene *Lr26*, all derived from chromosome arm 1RS of Petkus rye; *Pm17* came from 1RS of Insave rye; *Pm7* derived from 2RL of Rosen rye and *Pm20* from 6RL of Prolific rye [5]. However, the resistance genes have been in strong selection pressure owing to continual variance of the virulent isolates even as a serious epidemic of powdery mildew during 1990–1991 in China due to the invalidation of *Pm8* [6], and what is more *Pm7* and *Pm17* were also no longer effective. Thus, to meet the challenge of the rapid loss of resistance, it is essential to continually develop new resistance germplasm and identify novel resistance gene sources from other rye genotypes against new virulent isolates.

As a cross-pollinated crop, rye contained significant genetic diversity within and between cultivars. Winter rye cultivar German White showed immune to different virulent *Bgt* isolates at the seedling stage and the composite *Bgt* isolates prevalent in northern China at the adult stage [7]. Winter wheat cultivar Xiaoyan 6, as a famous cultivar and founder parent of wheat, has been widely grown for the past 25 years [5] and developed more than 50 wheat cultivars in China [8], but showed susceptible to powdery mildew at both seedling and adult stages. To improve its powdery mildew resistance, we transferred chromosomes or chromosome segments of German White rye into Xiaoyan 6 by distant hybridization, chromosome manipulation and self-cross for many generations since 1995, and several chromosome translocation lines [7,9] and substitution line [10] were developed and characterized. Among the progenies of Xiaoyan 6 and German White, a new wheat-rye 6R chromosome addition line WR49-1 showed a high level of resistance to powdery mildew currently prevailing in northern China during the whole growing stage. This study was aimed at developing chromosome addition line, determining the chromosome composition of WR49-1 using molecular cytogenetic methods, characterizing its resistance to powdery mildew using different isolates of the pathogens, and evaluating its agronomic performance.

## Materials and Methods

### Plant materials

The winter wheat cultivar Xiaoyan 6 was derived from hybridization of common wheat and *Thinopyrum ponticum* (2n = 10x = 70) [8]. Wheat-rye line WR49-1 was produced by crossing Xiaoyan 6 with the winter rye cultivar German White. The cultivars Mingxian 169 and Huixianhong were used as susceptible controls and spreader of the powdery mildew. To determine the powdery mildew resistance in WR49-1, a differential set containing 38 wheat cultivars/lines with documented *Pm* genes or gene combinations were used as controls, including TAM104/Thatcher with *Pm20* derived from rye chromosome arm 6RL, CI14189 with *Pm*7 from 2RL, Kavkaz with *Pm8* and Amigo with *Pm17* both from 1RS. Twenty-three single-pustule-derived powdery mildew virulent isolates was used to test different wheat genotypes in the same way to differentiate their reaction patterns.

Total genomic DNA isolated from wheat cultivar Chinese Spring (CS, ABD genomes) [11] was used as blocking DNA in genomic *in situ* hybridization (GISH) and multicolor fluorescence *in situ* hybridization (mc-FISH) detection of WR49-1. The following lines were used as controls to detect rye chromatin in WR49-1 by PCR (polymerase chain reaction) analysis, including two T1BL·1RS wheat-rye chromosome translocation lines Lovrin 10 and Lovrin 13 [4], two triticale lines 10R2-193-2 and 10R2-194-2 (AABBRR), three wheat-rye lines (WR41-1, WR81 and WR91) derived from 'Xiaoyan 6 × German White' and previously identified by using GISH and mc-FISH, a set of wheat-rye disomic addition lines (DA1R to 7R) of 'CS × Imperial' kindly supplied by Dr S. Reader (John Innes Centre, Norwich, UK).

### Development of wheat-rye chromosome addition line

The following procedure was used for the production of resistant wheat-rye addition line WR49-1. Wheat cultivar Xiaoyan 6 was crossed with rye cultivar German White in 1996 and 16–18 d following pollination, inflorescences were collected and stored at 4°C for 48–72 h. Hybrid embryos were dissected from young panicles and 12 plants were obtained by embryo rescue on MS medium. Following chromosome doubling with a solution containing 0.05% colchicine, 1.5% dimethylsulfoxide, and 5% MS medium [12], six amphidiploid plants with somatic cell chromosome numbers 2n = 56 were synthesized and tested for resistance to a composite of *Bgt* isolates prevalent in northern China. Six plants with powdery mildew resistance were selected to make backcross with the wheat parent Xiaoyan 6. The plants of BC_1_F_1_ were continually screened for resistance to powdery mildew, and the resulting resistant plants as females were continually back-crossed with Xiaoyan 6. The BC_2_F_1_ plants were again tested for resistance to powdery mildew and karyotyped by cytological examination. Resistant plants of putative rye monosome addition lines with chromosome numbers 2n = 43 were allowed to self-pollinate. The plant with somatic cell chromosome number 2n = 44 was selected and continually self-pollinated for an additional five generations. During development, each individual plant was selected based on resistance to powdery mildew, wheatlike plant type and high seed set, and then karyotyped by cytological examination. Finally, a fertile and genetically stable line with homogeneous resistance, designated as WR49-1, was selected.

### GISH analysis

GISH technique was used for detection of rye chromatin in wheat-rye line WR49-1. The mitotic chromosomes of root tips cells of WR49-1 were prepared and observed by the method of An et al. [10]. Total genomic DNA of German White rye was labeled with fluorescein-12-dUTP by nick translation method and used as a probe, detection and visualization were performed as previously described [7].

### Sequential multicolor FISH analysis

FISH analysis was used to characterize rye and wheat chromosomes in WR49-1. After rinsing the GISH hybridization probes signals, mc-FISH was conducted by using two highly repeated DNA sequences p*As1* (or p*HvG38*) labeled with digoxigenin-11-dUTP and p*Sc119*.*2* labeled with biotin-11-dUTP, respectively [13]. Detection and visualization were done as described above. Two repeated sequences p*Sc119*.*2* and p*As1* as probes enabled all the R-genome chromosomes of rye, 17 of 21 chromosome pairs of hexaploid wheat to be distinguished from each other [14], whereas probe p*As1* combined with p*HvG38* could identify all 21 wheat chromosome pairs [15].

### Multicolor-GISH (mc-GISH) analysis

Total genomic DNA was isolated from young leaves of *T*. *Urartu* (2n = 2x = 14, AA), *Aegilops speltoides* (2n = 2x = 14, BB) and *Ae*. *Tauschii*, respectively [11]. Total genomic DNA of rye and *T*. *urartu* were labeled with fluorescein-12-dUTP, and total genomic DNA of *Ae*. *tauschii* was labeled with Texas-red-5-dUTP, while total genomic DNA of *Ae*. *speltoides* was used for blocking [16]. Detection and visualization were performed as previously described [7].

### PCR analysis

Seven primer pairs were used to identify rye chromosome in WR49-1: one EST-STS (expressed sequence tag-sequence tagged site) marker CGG143 specific for chromosome 6R of Imperial rye, and six EST-SSR (simple sequence repeat) markers specific for chromosome arm 6RL of Imperial rye, including SWES78, SWES206, SWES231, CGG23, CGG59 and DUPW111 [17]. PCR reactions were performed, and products were separated and visualized as described.

### Assessment of the powdery mildew resistance

Reactions of WR49-1 to 23 single-pustule-derived powdery mildew isolates at the seedling stage were tested as described by An et al. [7]. Xiaoyan 6, German White, Mingxian 169, Huixianhong and a set of 38 wheat cultivars/lines carrying documented *Pm* genes or gene combinations (Table 1) were used as controls. When the susceptible controls Mingxian 169 and Huixianhong showed fully disease symptoms at the first leaf, infection types (IT) of each plant for each isolate was recorded according to a 0–4 scale, plants with IT 0–2 were considered resistant and those with IT 3–4 susceptible [18].

**Table 1:** Seedling disease responses of wheat-rye line WR49-1, its rye parent German White, wheat parent Xiaoyan 6, and 38 different wheat genotypes possessing documented powdery mildew resistance (*Pm*) gene(s) or gene combinations to 23 different *Blumeria graminis* f. sp. *tritici* (*Bgt*) isolates.

Cultivar/line	*Pm* gene	*B*. *graminis* f. sp.*tritici* isolates																						
		E01	E02	E05	E06	E07	E09	E11	E13	E15	E16	E17	E18	E20	E21	E23-(1)	E23-(2)	E26	E30-(1)	E30-(2)	E31	E32	E49	E50
German White	Unknown	R	R	R	R	R	R	R	R	R	R	R	R	R	R	R	R	R	R	R	R	R	R	R
Xiaoyan 6	—	S	S	S	S	S	S	S	S	S	S	S	S	S	S	S	S	S	S	S	S	S	S	S
WR49-1	Unknown	R	R	R	R	R	R	R	R	R	R	R	S	S	S	R	R	R	R	R	R	R	S	R
Mingxian 169	—	S	S	S	S	S	S	S	S	S	S	S	S	S	S	S	S	S	S	S	S	S	S	S
Huixianhong	—	S	S	S	S	S	S	S	S	S	S	S	S	S	S	S	S	S	S	S	S	S	S	S
TAM104/Thatcher	20	S	R	R	R	S	S	R	R	R	S	R	S	R	S	S	R	R	R	R	S	S	S	R
Kavkaz	8	R	S	S	S	S	S	S	S	S	S	S	S	S	S	R	S	S	S	S	S	R	R	R
CI14189	7	S	S	S	S	S	S	S	S	S	S	S	S	S	S	S	S	S	S	S	S	S	S	S
Amigo	17	S	S	S	R	S	S	S	S	S	S	S	S	S	S	S	S	S	S	S	S	S	S	S
Funo	~	S	S	S	S	S	S	S	S	S	S	S	S	S	S	S	S	S	S	S	S	S	S	S
Ulka/8cc	2	R	R	R	R	R	R	R	R	R	R	R	S	S	S	R	R	R	R	R	R	S	R	R
Maris Huntsman	2+6	R	R	R	R	R	R	R	R	R	R	R	S	S	S	R	R	R	R	R	R	S	R	R
Baimianmai3	4+8	R	R	R	R	R	R	R	R	S	R	R	S	S	S	R	R	R	R	R	S	R	R	R
Xiaobaidongmai	XBD	R	S	R	S	S	R	S	S	S	R	S	R	R	S	S	R	R	R	R	R	S	R	R
Chancellor	—	S	S	S	S	S	S	S	S	S	S	S	S	S	S	S	S	S	S	S	S	S	S	S
Axminster/8cc	R	S	S	S	R	S	S	R	S	S	S	S	S	S	S	S	S	S	S	S	S	S	S	S
Asosan/8cc	3a	S	S	S	S	S	S	S	S	S	S	S	S	S	S	S	S	S	R	R	S	S	S	S
Chul/8cc	3b	S	R	S	R	R	S	R	S	R	S	S	S	S	S	S	S	S	S	S	S	S	S	S
Sonora/8cc	3c	S	S	S	S	S	S	S	S	R	S	S	S	S	S	S	S	S	S	S	S	S	S	S
Kolibri	3d	R	S	S	R	S	S	R	R	S	S	S	S	S	S	R	S	S	R	R	S	S	R	S
Mich.Amber/8cc	3f	S	S	S	S	S	S	S	S	S	S	S	S	S	S	S	S	S	S	S	S	S	S	S
W150	3e	S	S	S	S	S	S	S	S	S	S	S	S	S	S	S	S	S	S	S	S	S	S	S
Khapli/8cc	4a	R	R	R	R	R	R	R	R	S	R	R	S	S	S	R	R	R	R	R	S	R	R	R
Armada	4b	R	R	R	R	R	R	R	R	R	R	R	S	S	S	R	R	R	R	R	S	R	R	R
Hope/8cc	5a	S	S	S	S	S	S	S	S	S	S	S	S	S	S	S	S	S	S	S	S	S	S	S
Aquila	5b	R	R	R	R	R	R	R	R	R	R	R	S	S	S	R	R	R	R	R	R	S	R	R
Coker 983	5+6	S	S	R	S	S	S	S	S	R	S	S	S	S	S	S	S	R	R	R	S	S	S	S
Timgalen	6	S	S	S	—	S	S	S	S	S	S	S	S	S	S	S	S	S	S	S	S	S	S	S
Coker 747	6	S	S	S	S	S	S	S	S	S	S	S	S	S	S	S	S	S	S	S	S	S	S	S
Wembley	12	R	R	R	R	R	R	R	R	R	R	R	R	R	R	R	R	R	R	R	R	R	R	R
R4A	13	R	R	S	R	R	R	R	S	S	R	R	—	R	R	S	R	R	S	R	R	R	S	S
Brigand	16	R	R	R	R	R	R	R	R	R	R	R	R	R	R	R	R	R	R	R	R	R	R	R
XX186	19	S	S	S	S	S	S	S	S	S	S	S	S	S	S	S	S	S	S	S	S	S	S	S
DS6 V(6A)	21	R	R	R	R	R	R	R	R	R	R	R	R	R	R	R	R	R	R	R	R	R	R	—
Chiyacao	24	R	R	S	S	R	R	R	S	S	R	R	S	S	S	S	R	R	S	R	S	S	S	S
Maris Dove	2+MLD	R	R	R	—	R	R	R	R	R	R	R	S	S	S	R	R	R	R	R	R	S	R	R
Mission	4b+5b	R	R	R	R	R	R	R	R	R	R	R	S	S	S	R	R	R	R	R	S	R	R	R
Normandie	1+2+9	S	S	S	S	S	S	S	S	S	S	S	S	S	S	S	S	S	S	R	S	S	S	S
5P27	30	R	R	R	—	R	R	R	R	R	R	R	S	S	S	R	R	R	R	R	S	R	R	R
81–7241	4c	R	R	R	R	R	R	R	R	R	R	R	S	S	S	R	R	R	R	R	S	S	R	R
NCA5	25	S	S	S	R	S	S	R	S	R	S	S	S	S	S	S	S	S	R	R	S	S	R	R
NCD7	34	S	R	R	S	S	S	R	S	R	S	R	S	S	S	S	S	R	R	R	S	S	S	R
NCD3	35	R	R	R	S	S	S	S	S	R	R	R	R	S	S	R	R	R	R	R	R	R	R	R

Infection types 0–2 were resistant (R) and 3–4 were susceptible (S) as described by Si et al. (1992). R: resistant host reaction, S: susceptible host reaction, and—: no data.

The adult plant reaction test was repeatedly conducted in 2011–2014 growing seasons at Luancheng Agro-Ecological Experimental Station, Chinese Academy of Sciences, Shijiazhuang, China. The composite *Bgt* isolates collected from northern China [7] was used as inoculums. The reactions to powdery mildew were tested on WR49-1, its parents and controls for three replicates in field condition. Disease reaction was recorded on a 0–9 scale, where 0–4 was considered resistant and 5–9 susceptible [19].

### Evaluation of agronomic performance

Evaluation of agronomic performance of WR49-1 was carried out as described by An et al. [7]. WR49-1 and its parents Xiaoyan 6 and German White were hand planted. Each plot consisted of six 1.5-m-longs, 30 seeds per row with inter-row spacing of 0.25 m. The plots were arranged in a randomized block design with three replications. At the physiology maturity stage, 15 whole plants were manually harvested from the centre of the three inner rows. Measurement and counting were done on plant height, spike length, spike number per plant, spikelet number per spike, sterile spikelet number per spike, kernel number per spike, thousand-kernel weight (TKW) and grain yield per plant.

## Results

### GISH analysis of WR49-1

**Fig 1:**
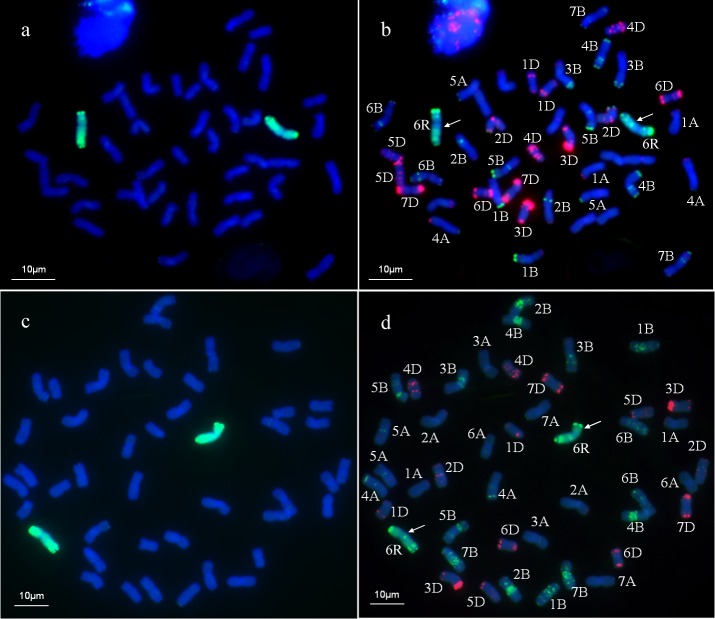
Genomic *in situ* hybridization (GISH) and sequential multicolor fluorescence *in situ* hybridization (mc-FISH) analysis of the wheat-rye chromosomes addition line WR49-1. **a** and **c** using rye genomic DNA as a probe and Chinese Spring DNA as a blocker, GISH detection of WR49-1 clearly show bright green hybridization signals evenly distributed on rye chromosomes, the wheat chromosomes were counterstained with 4, 6-diamidino-2-phenylindole (DAPI) (blue). **b** Sequential mc-FISH on the same metaphase after GISH analysis (**a**) of WR49-1 by p*As*1 (red) and p*Sc*119.2 (green) simultaneously. **d** mc-FISH on the same metaphase after GISH analysis (**c**) of addition line WR49-1 by p*As*1 (red) and p*HvG*38 (green) simultaneously. Arrows note one pair of added 6R chromosomes.

**Fig 2:**
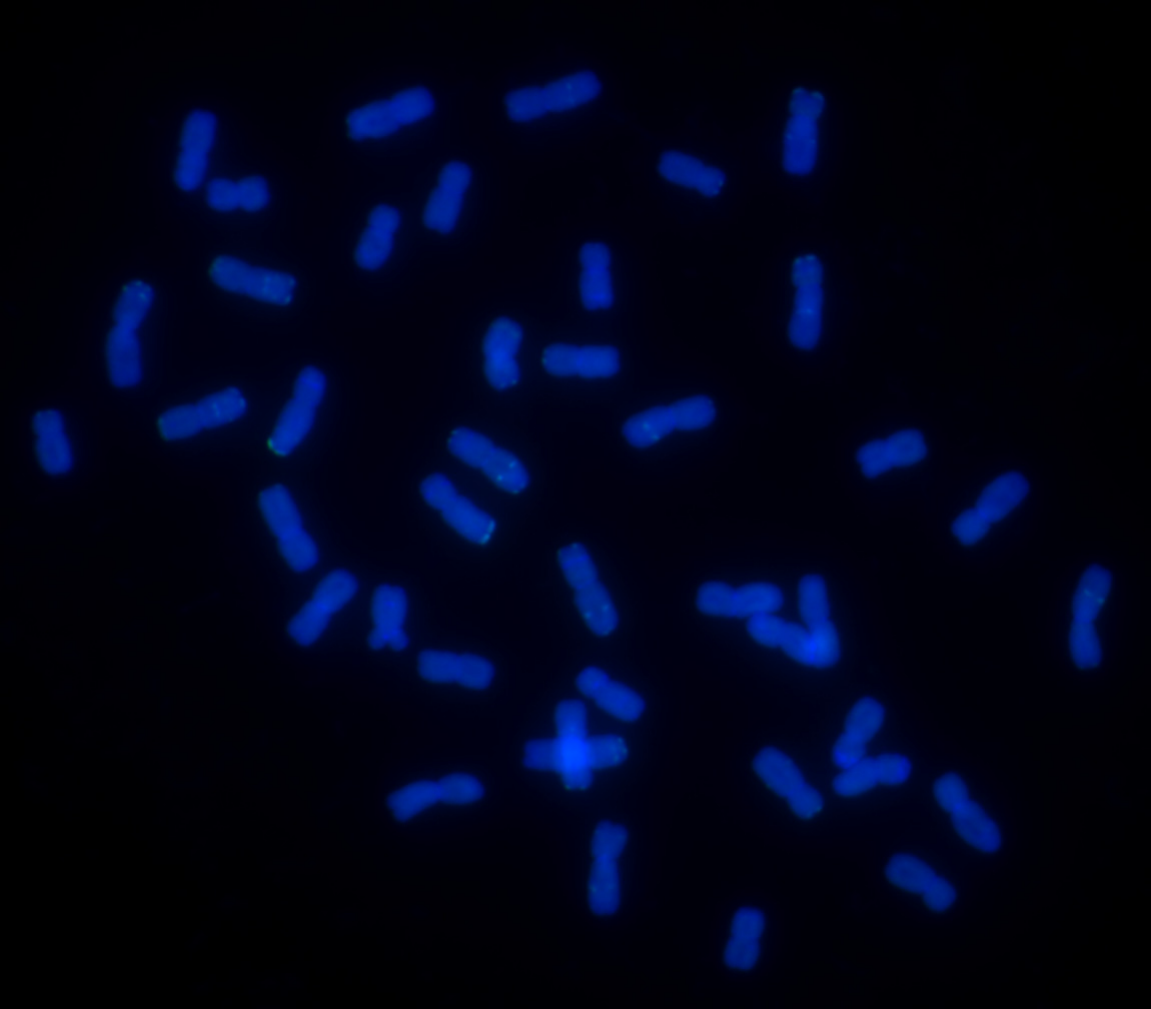
GISH analysis of wheat cultivar Xiaoyan 6. Using rye genomic DNA as a probe and Chinese Spring DNA as a blocker, GISH detection indicated that no rye chromosome or chromosome segment existed in Xiaoyan 6, the wheat chromosomes were counterstained with DAPI (blue).

The GISH analysis was used to test the presence of rye chromosomes in WR49-1. The result indicated that WR49-1 had 44 chromosomes, among which one pair of intact chromosomes displayed bright-green hybridization signals and demonstrated that it came from the rye parent German White, while the other 42 chromosomes showed only the blue signals, indicating that they were derived from the wheat parent Xiaoyan 6 (Fig 1a and 1c), since GISH detection showed that no rye chromosomes or chromosome segment existed in Xiaoyan 6 (Fig 2). GISH analysis for progeny plants in five consecutive selfing generations of WR49-1 further confirmed this result, indicating that WR49-1 was a genetically stable wheat-rye addition line.

### Mc-FISH analysis of WR49-1 after GISH

After GISH analysis, mc-FISH with three probes, p*Sc119*.*2* and p*As1* (or p*HvG38*) was performed to characterize rye and wheat chromosomes in WR49-1. The additional rye chromosome in WR49-1 had five specific hybridization bands when labeled with p*Sc119*.*2*, including one strong terminal band and one faint subterminal band on the short arm, as well as two strong intercalary bands and one faint terminal band on the long arm (Fig 1b). The two rye chromosomes did not bear p*As1* hybridization signals (Fig 1b). Thus, the additional rye chromosome was identified as 6R [14]. Using p*HvG38* and p*As1* as probes, all 21 pairs of wheat chromosomes were discriminated from each other as previously described [15] (Fig 1d). Based on the p*Sc119*.*2*, p*As1*, and p*HvG38* diagnostic banding pattern, WR49-1 was identified as a 6R chromosome addition line.

### Mc-GISH analysis of WR49-1

**Fig 3:**
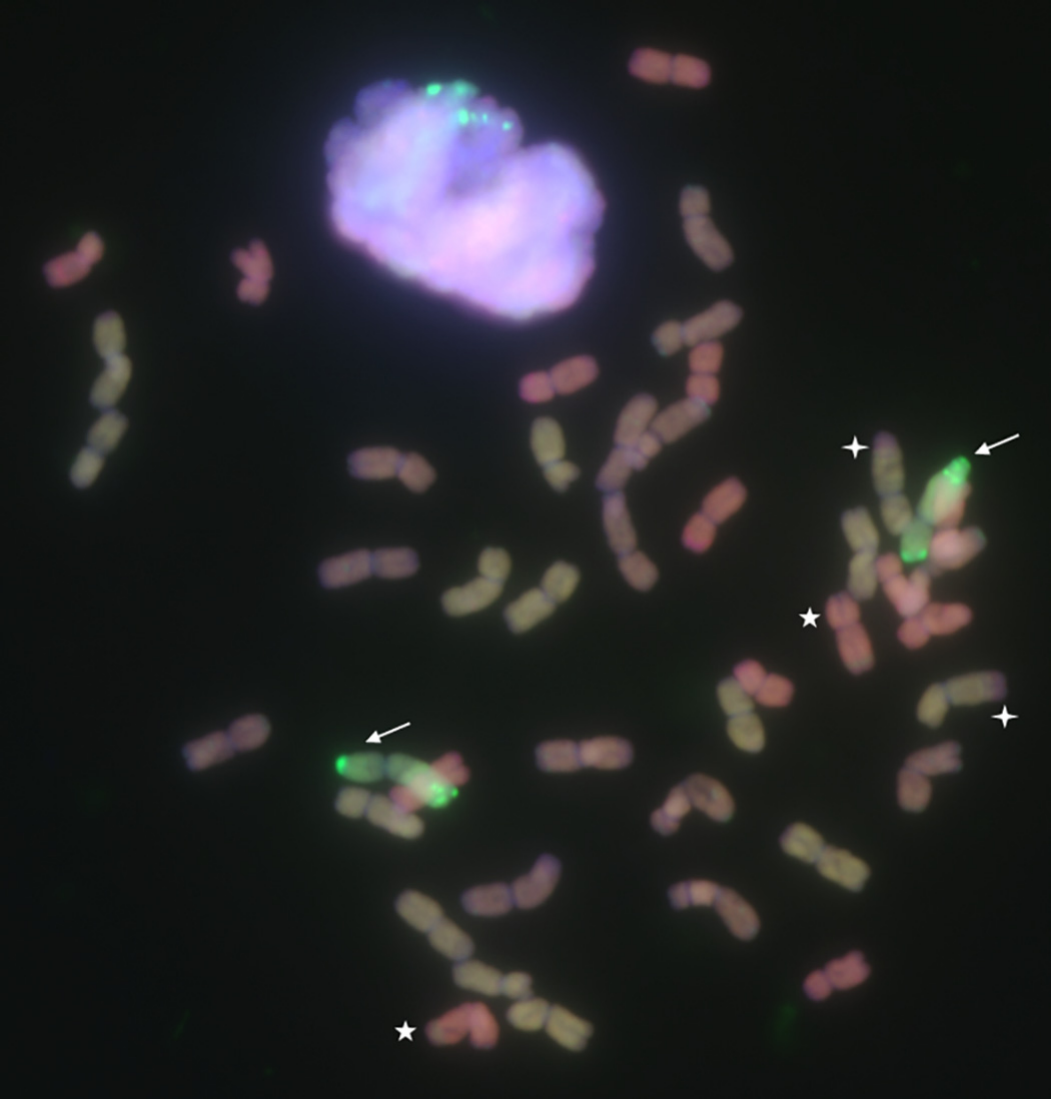
Multicolor-genomic *in situ* hybridization (mc-GISH) analysis of the wheat-rye chromosome addition line WR49-1. Yellow-green, brown or gray and pink or red are A-genome chromosomes, B-genome chromosomes and D-genome chromosomes, respectively, and bright-green is added R-genome chromosomes. Arrows indicate one pair of added rye chromosomes. Two small translocations between B and D, B and A chromosomes were also detected in WR49-1, respectively. Asterisks note two small B-genome chromosome segments are translocated to D-genome chromosomes, and crosses note other two small B-genome chromosome segments are translocated to A-genome chromosomes.

To further confirm the genomic identity of the chromosomes in WR49-1, mc-GISH analysis was done by using the total genomic DNA of the various diploid progenitors of common wheat and rye as probes or blockers. The A-, B-, and D-genome chromosomes were detected by yellow-green fluorescence, brown or gray, and red or pink fluorescence, respectively as a result of cross-hybridization by the different genomic probes [16], while the rye chromatin were detected by green fluorescence signals. The result showed that WR49-1 had 14 genome chromosomes for A-, B-, and D-genome each and one pair of rye chromosomes, indicating WR49-1 was a rye chromosome addition line (Fig 3). Meanwhile, two small translocations between B- and D-, B- and A- genome chromosomes were detected in WR49-1. Two small B-genome chromosome segments were translocated to the long arms of D-genome chromosomes (Fig 3, asterisks) and other two small B-genome chromosome segments were translocated to the long arms of A-genome chromosomes (Fig 3, crosses). No other gross translocation was detected in WR49-1.

### PCR analysis of WR49-1

**Fig 4:**
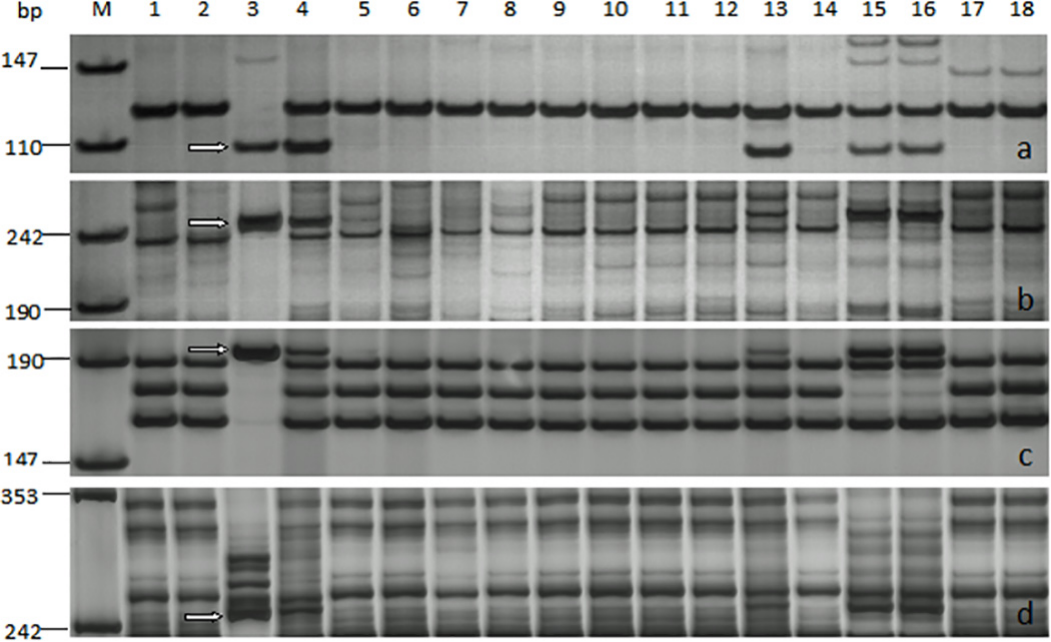
PCR amplification of expressed sequence tag (EST)-based markers. CGG143 (**a**) specific for rye chromosome 6R, and SWES78 (**b**), SWES206 (**c**) and SWES231 (**d**) specific for 6RL, respectively, in wheat-rye lines and controls for detection of 6R in WR49-1. The 110, 260, 200 and 260 bp bands indicate the diagnostic DNA fragments specific for 6R or 6RL, respectively. Lanes *M* marker pUC18/*Msp*I, *1* Chinese Spring, *2* Xiaoyan 6, *3* German White, *4* WR49-1. *5* WR41, *6* WR81 and *7* WR91 lines derived from 'Xiaoyan 6 × German White' without 6R identified by multicolor fluorescence *in situ* hybridization (mc-FISH). *8* to *14* are 1R-7R addition lines of 'Chinese Spring × Imperial', respectively. *15* and *16* are triticale lines 10R2-193-2 and 10R2-194-2 (AABBRR). *17* Lovrin 10 and *18* Lovrin 13 are T1BL·1RS chromosome translocation lines.

Among the seven EST-based markers specific for chromosome 6R or 6RL of Imperial rye used, CGG143, SWES78, SWES206 and SWES231 could amplify about 110, 260, 200 and 260 bp DNA fragments from German White rye, respectively (Fig 4a, 4b, 4c and 4d). The corresponding DNA fragments were also respectively detected in 6R disomic addition line of 'CS × Imperial' and two triticale lines 10R2-193-2 and 10R2-194-2 (AABBRR); but not in the other addition lines of 'CS × Imperial', and the other control genotypes, including the three lines of 'Xiaoyan 6 × German White' (WR41-1: 4R translocation line, WR81: T1BL·1RS translocation line and WR91: 2R (2D) substitution line), two T1BL·1RS translocation lines (Lovrin 10 and Lovrin 13), as well as CS and Xiaoyan 6. The results indicated the four markers can be used for detecting chromosome 6R of German White rye. WR49-1 amplified the specific bands consistent with German White rye (Fig 4a, 4b, 4c and 4d), indicating it contain DNA regions specific for chromosome 6R of German White.

Based on comprehensive analyses of GISH, mc-FISH, mc-GISH and PCR, WR49-1 was determined to be a 6R chromosome disomic addition line involving two small translocations between B- and D-, B- and A-genome chromosomes and therefore its chromosome composition was 2n = 44 = 21″W+1″R (6R).

### Response to powdery mildew of WR49-1

**Fig 5:**
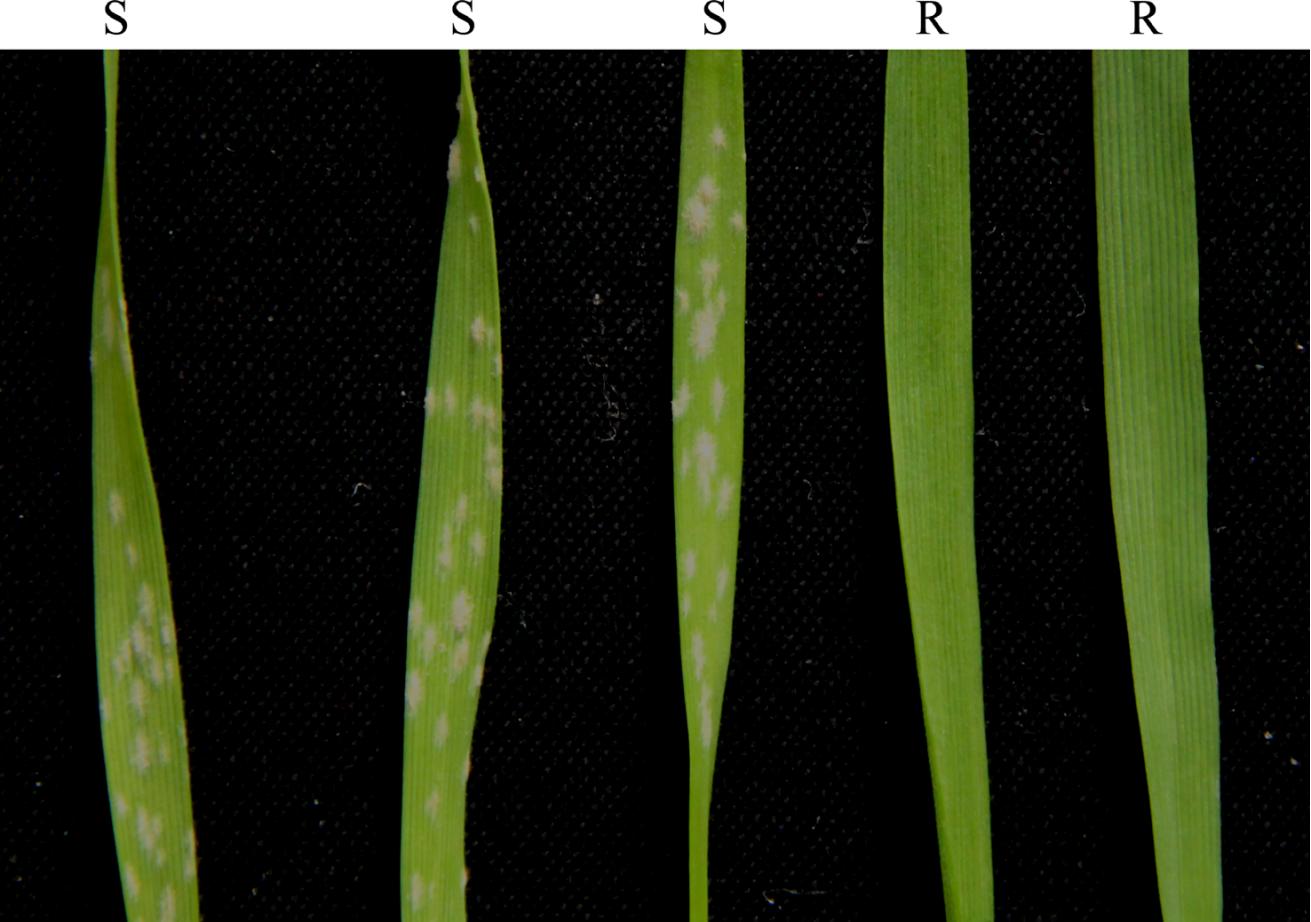
Disease responses of Mingxian 169, Huixianhong, Xiaoyan 6, German White and WR49-1 (*from left to right*) to isolate E09 of *Blumeria graminis* f. sp. *tritici* (*Bgt*) at the seedling stage. R indicates resistant host reaction, and S indicates susceptible host reaction.

The seedlings of WR49-1, German White rye, Xiaoyan 6, Mingxian 169, Huixianhong and 38 wheat genotypes carrying documented *Pm* gene or gene combinations were tested for their reactions to 23 *Bgt* isolates (Table 1). The susceptible controls Mingxian 169 and Huixianhong were all highly susceptible with IT 4. German White was immune to all the 23 isolates with IT 0, while Xiaoyan 6 was susceptible to these isolates, including isolate E09 (Fig 5). WR49-1 was resistant to 19 of 23 isolates, except for isolates E18, E20, E21 and E49 (Table 1). By comparing the reaction patterns between WR49-1 and the wheat genotypes used in this study, it was found that WR49-1 was significantly different from the 38 genotypes with documented *Pm* genes or gene combinations (Table 1). For example, WR49-1 had significantly different reaction patterns on 8 isolates with TAM104/Thatcher carrying *Pm20* derived from 6RL. WR49-1 was highly resistant to isolates E01, E07, E09, E16, E23-(1), E31 and E32, but susceptible to E20, whereas TAM104/Thatcher showed reverse reaction patterns to these 8 isolates. Therefore, the resistance to powdery mildew by WR49-1 appeared to be controlled by new *Pm* gene(s) different from documented *Pm* genes or gene combinations.

In three consecutive wheat growing seasons, WR49-1, German White and Xiaoyan 6, along with six controls, including Mingxian 169, Huixianhong, TAM104/Thatcher with *Pm20*, Kavkaz with *Pm8*, CI14189 with *Pm7* and Amigo with *Pm17*, were tested with the mixture of *Bgt* isolates collected from northern China. Xiaoyan 6 and the five controls, Kavkaz, CI14189, Amigo, Mingxian 169 and Huixianhong, showed all susceptible with IT from 8 to 9, whereas German White developed no mildew symptoms with IT 0. WR49-1 and TAM104/Thatcher with *Pm20* showed IT 1, therefore, were considered as highly resistant [19].

### Agronomic character of WR49-1

**Fig 6:**
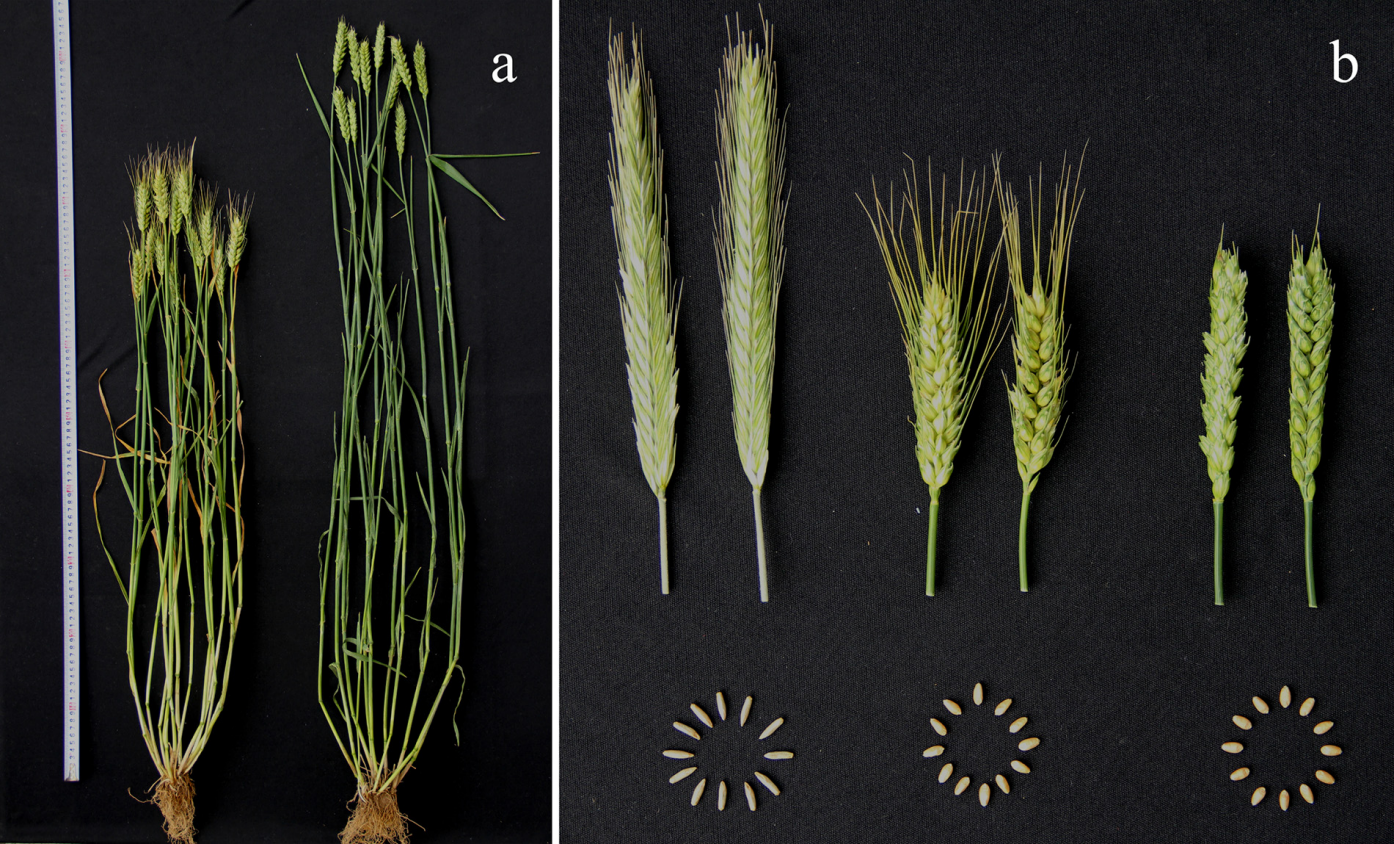
Morphology performance of wheat-rye chromosome addition line WR49-1 and its parents. **a** Plants of parent Xiaoyan 6 and WR49-1 (*left and right*). **b** Spikes and seeds of German White, Xiaoyan 6, and WR49-1 (*from left to right*).

Neither morphology nor cytology segregation was observed after five consecutive generations of selfing for WR49-1. The morphology of WR49-1 was similar to common wheat: compact plant type, short awn and plump red seeds (Fig 6a and 6b). The trait values of plant height, average spike length, spikelet number per spike and grain yield per plant for WR49-1 were all significantly higher than its wheat parent Xiaoyan 6 but less than its rye parent German White. WR49-1 exhibited higher kernel number per spike (KNS) but lower thousand-kernel weight (TKW) than Xiaoyan 6 (*P*<0.05), whereas no significant difference with German White (Table 2). The spike number per plant of WR49-1 were less than both parents (*P*<0.05). WR49-1 possessed superior fertility than both parents for its desirable performance on sterile spikelet number per spike (Table 2).

**Table 2:** The agronomic traits of wheat-rye line WR49-1 and its parents Xiaoyan 6 and German White rye.

Traits	WR49-1	Xiaoyan 6	German White
Plant height (cm)	78.6±2.1^b^	64.1±2.4^c^	146.7±7.1^a^
Spike length (cm)	7.7±0.5^b^	6.0±0.0^c^	13.3±1.3^a^
Spike number per plant	8.7±2.2^c^	10.0±1.5^b^	34.3±7.9^a^
Spikelet number per spike	21.2±1.0^b^	18.7±1.0^c^	43.1±2.8^a^
Sterile spikelet number per spike	0.1±0.3^c^	1.4±0.5^b^	2.6±1.1^a^
Kernel number per spike	70.6±7.9^a^	39.0±8.6^b^	70.1±8.4^a^
Thousand—kernel weight (g)	30.2±0.8^b^	37.7±2.0^a^	28.8±0.7^b^
Grain yield per plant (g)	11.3±1.4^b^	8.6±1.9^c^	49.7 ±11.8^a^

For a specific trait, the values followed by the same letters were not different significantly at the 0.05 probability level according to the LSD test.

## Discussion

Chromosome 6R of rye possessed many genes for favorable traits, such as resistances to powdery mildew [3], stripe rust [20], cereal cyst nematode (*Heterodera avenae*) [21], *H*. *filipjevi* nematode [22], Hessian fly (*Mayetiola destructor*) [23], as well as tolerance to aluminum [24] and drought [25], which were useful for wheat improvement. Xiaoyan 6 had high-yielding, good bread-making quality with HMW-GS (high molecular weight glutenin subunits) *1Bx 14* and *1By 15*, early maturity, stress tolerance and wide adaptation, but showed vulnerable to powdery mildew. In order to improve the powdery mildew resistance of Xiaoyan 6 and in view of the multiple potentially useful genes on rye chromosome 6R, we conducted wide hybridization and chromosome manipulation between Xiaoyan 6 and rye cultivar German White, developed and characterized a new wheat-rye 6R disomic addition line WR49-1.

WR49-1 conditioned absolute resistance against the composite of *Bgt* isolates prevalent in northern China at the adult stage and showed high resistance to 19 of 23 *Bgt* isolates at the seedling stage. It had different reaction patterns with *Pm7*, *Pm8*, *Pm17* and *Pm20* derived from rye (Table 1) and a wider array of resistance to *Bgt* isolates than TAM104/Thatcher with *Pm20* from 6RL. WR49-1 may possess new *Pm* gene(s) and is an additional source for powdery mildew resistance, thus can be used in broadening mildew resistance in wheat. Additionally, it is necessary for identifying the resistance genes in WR49-1 to conduct genetic and molecular mapping studies.

At present, more than 70 formally designated *Pm* genes at 49 loci and more than 20 temporarily named *Pm* genes have been reported in common wheat and its wild relatives [26,27], rye cataloged four genes *Pm7*, *Pm8*, *Pm17* and *Pm20* [3,28–30]. Of those, dominant resistance gene *Pm8* from T1BL·1RS translocation line, *Pm17* from T1AL·1RS translocation line, and *Pm20* from T6BS·6RL translocation line showed highly resistant to 5, 1 and 13 of 23 *Bgt* isolates tested in this study, respectively. Currently, the cultivars with *Pm8* and *Pm17* have already successively lost the resistance to powdery mildew [6]. Another dominant resistance gene *Pm7* from T4BS·4BL-2RL translocation line, also exhibited highly susceptible to all the 23 *Bgt* isolates tested (Table 1).

In a previous study, using a set of isogenic wheat-rye addition, substitution and translocation lines, their resistance patterns to powdery mildew were compared and analyzed, and the result indicated that rye chromosomes 6R conditioned absolute resistance to all powdery mildew isolates tested and resistance gene was located on 6RL [31], which was in accordance with another study for chromosomes 6R [32]. Wang et al. [33] located a powdery mildew resistance locus on 6RL of rye cultivar Jingzhouheimai.

The gene *CreR* conferred very strong resistance to the cereal cyst nematode and was available to wheat breeders in the form of a Chinese Spring 6R (6D) substitution line, had located on 6RL of rye, further was mapped 3.7 cM distal from the RFLP locus, *XksuF37* [21,34]. *H*. *filipjevi* was a newly identified pathogenic species of cereal cyst nematode that has severely damaged wheat in central China. A two-year field test demonstrated that the substitution line 6R (6D) was also highly resistant to the nematode [22]. A Hessian fly resistance gene *H25* was located on the 6RL telocentric chromosome of Balbo rye and transferred into hexaploid wheat via *X*-irradiation-induced terminal and intercalary chromosomal translocations [23].

Curtis and Lukaszewski [35] reported on the capability of a rye gene from 6RL to restore male fertility in wheat with the *T*. *timopheevii* cytoplasm. Using a set of CS-Imperial disomic addition lines, QTLs (quantitative trait locus) controlling agro-physiological indicators of drought tolerance were also detected on chromosomes 6R [25]. A loci *Alt1* controlling aluminum tolerance was located on 6RS of rye and two isozyme loci linked to *Alt1* gene was also identified on 6RS [24].

Resistance genes to wheat stripe rust were also located on 6RL of rye [20]. Our results showed that Germany White rye was highly resistant to American stem rust races 72–41, 72, TNMK, QCC-2, 370C and 81AC46-2, and WR49-1 to 72–41, TNMK, QCC-2 and 81AC46-2. Furthermore, both Germany White rye and WR49-1 displayed highly resistance to the virulent isolate R-46 of sharp eyespot (*Rhizoctonia cerealis*), which may improve the progress of sharp eyespot resistance breeding in wheat. However, for different resistances of WR49-1, more genetic and molecular mapping studies are needed to identify the resistance genes and determine gene interactions.

The alien chromosome disomic addition lines were not only valuable genetic resources for studying the origin and evolution of species, genetic relationships between different genomes, gene interactions and expression, but also important intermediate materials for developing substitution lines and translocation lines [36]. To date, several wheat-rye 6R addition lines had been developed and identified, including CS-Imperial, Holdfast-King II, CS-King II, and Kharkov-Dakold additional lines [36]. Using amphidiploids and backcross strategy, wheat-rye disomic addition lines were obtained [37]. Combining anther culture with chromosome doubling, a 6R disomic addition line was developed [38]. Nevertheless, few wheat-rye lines involving chromosome 6R have been used in wheat improvement due to agronomic disadvantages. For example, T6BS·6RL translocation line TAM104 showed high level resistance to more than half *Bgt* isolates in China (Table 1), but had susceptibility to lodging and poor grain filling, rendering it unsuitable for wheat breeding [39]. The wide usefulness of wheat-alien hybrids depends, to some extent, on their wheat genetic backgrounds. In this study, the 6R chromosome addition line WR49-1 had a favorable wheat genetic background, possessed superior plump seeds and high spikelet number per spike and kernel number per spike (Fig 6a and 6b and Table 2).

Chromosome instability and genome rearrangements in wheat-rye addition lines have been well documented [40,41]. Among the set of CS-Imperial disomic addition lines, only 6R and 4R chromosomes displayed no variation [42]. In this study, progeny plants in five consecutive selfing generations of WR49-1 were confirmed no variations by GISH analysis, indicating that WR49-1 was a genetically stable wheat-rye addition line.

In addition to *in situ* hybridization techniques, PCR-based markers have also become a convenient and efficient tool to identify the presence of alien chromosomes and chromosomal segments in wheat [43,44]. In this study, four out of seven EST-based markers specific for chromosome 6R or 6RL of Imperial rye were identified to be useful for detecting chromosome 6R of Germany White rye; meanwhile, WR49-1 was confirmed to have 6R from Germany White (Fig 4a and 4b). These markers are being used in marker-assisted selection for more Xiaoyan 6-German White derivatives.

The wheat-rye chromosome addition, substitution and translocation lines were valuable source materials for development of more highly engineered introgression lines. Comparing with addition and substitution lines, wheat breeders preferred translocation lines in view of the smaller amount of alien genetic material, less linkage drag, regular meiotic behavior and even direct utilization [45]. To further practically use W49-1 in wheat improvement and fine mapping of the resistance locus, we have performed to induce chromosome variations by ^60^Coγ radiation, *ph1b*-induced homoeologous recombination, gametocidal chromosome originating from *Aegilops* [46], and backcrossing strategy with commercialized cultivars. Small segmental translocations, particularly intercalary translocations only with the target locus and without linkage drags, and introgression lines are being identified by 6R-specific markers, GISH, mc-FISH and mc-GISH approaches. Finally, fine translocation lines involving small fragment with desirable agronomic traits and the closely associated molecular markers with resistance gene(s) will be obtained and used in wheat molecular marker-assisted resistance breeding for powdery mildew.
